# Phenotypic Heterogeneity in Expression of the K1 Polysaccharide Capsule of Uropathogenic Escherichia coli and Downregulation of the Capsule Genes during Growth in Urine

**DOI:** 10.1128/IAI.00188-15

**Published:** 2015-06-15

**Authors:** Jane E. King, Hasan A. Aal Owaif, Jia Jia, Ian S. Roberts

**Affiliations:** a Faculty of Life Sciences, University of Manchester, Manchester, United Kingdom; b College of Applied Biotechnology, Al-Nahrain University, Baghdad, Iraq

## Abstract

Uropathogenic Escherichia coli (UPEC) is the major causative agent of uncomplicated urinary tract infections (UTI). The K1 capsule on the surface of UPEC strains is a key virulence factor, and its expression may be important in the onset and progression of UTI. In order to understand capsule expression in more detail, we analyzed its expression in the UPEC strain UTI89 during growth in rich medium (LB medium) and urine and during infection of a bladder epithelial cell line. Comparison of capsule gene transcription using a chromosomal *gfp* reporter fusion showed a significant reduction in transcription during growth in urine compared to that during growth in LB medium. When examined at the single-cell level, following growth in both media, capsule gene expression appears to be heterogeneous, with two distinct green fluorescent protein (GFP)-expressing populations. Using anti-K1 antibody, we showed that this heterogeneity in gene expression results in two populations of encapsulated and unencapsulated cells. We demonstrated that the capsule hinders attachment to and invasion of epithelial cells and that the unencapsulated cells within the population preferentially adhere to and invade bladder epithelial cells. We found that once internalized, UTI89 starts to produce capsule to aid in its intracellular survival and spread. We propose that this observed phenotypic diversity in capsule expression is a fitness strategy used by the bacterium to deal with the constantly changing environment of the urinary tract.

## INTRODUCTION

Uropathogenic Escherichia coli (UPEC) is the predominant causative agent of urinary tract infections (UTI), responsible for up to 90% of all cases that arise in healthy individuals (1). Typically the disease starts in the bladder (cystitis), but it can ascend the ureters to cause a much more serious disease of the kidneys (pyelonephritis), which can result in permanent renal damage and the risk of subsequent septicemia (2). Women are far more susceptible to UTI, and by their mid-20s, 40% of all females will have experienced a UTI, with 25% experiencing a recurrence within 6 months of infection despite antibiotic therapy (3). With the increased emergence of antibiotic-resistant UPEC, there is a need for new therapeutic targets in the treatment of UTI (4, 5). As a consequence, understanding the molecular basis of UTI will help in the identification of such targets and fuel development of new therapies.

The pathogenesis of E. coli UTI has been studied extensively with mice, in which a multistep pathogenic cycle of infection has been demonstrated (6). UPEC initially adhere to the superficial epithelial (umbrella) cells that line the luminal surface of the bladder. This adhesion is mediated primarily via the FimH adhesin on type 1 fimbriae, which interacts with a number of host proteins, including integrins and uroplakin proteins that coat the apical side of umbrella cells (7–9). A number of mechanisms have been identified for UPEC invasion (10), and once UPEC organisms are inside host cells, a subset can escape expulsion and move into the cytoplasm, where they replicate to form intracellular bacterial communities (IBC), which have similarities to biofilm communities (6, 11). During an infection the IBC develop prior to killing the infected cell and spreading to adjacent epithelial cells, and these cycles of IBC formation and cell killing are responsible for the tissue damage and pathology typical of cystitis (11). The observation that IBC have been seen inside voided urothelial cells from human patients would suggest that a similar cycle exists in human infections (12).

**FIG 1:**
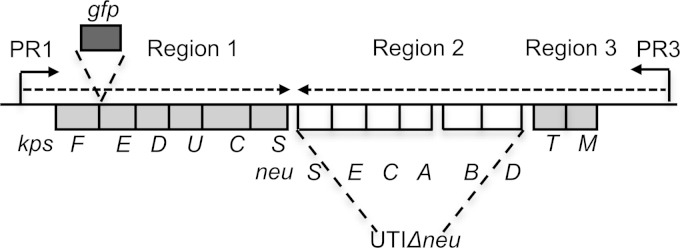
Genetic organization of the K1 capsule gene cluster. The gene cluster is composed of three regions. Region 1 (*kpsFEDUCS*) and region 3 (*kpsMT*) are conserved throughout group 2 capsules and encode proteins involved in the transport of the polysaccharide to the cell surface. Region 2 is serotype specific and in this case contains the genes responsible for the synthesis of the K1 polysaccharide. Dashed arrows indicate the major transcripts from promoters PR1 and PR3. The site of insertion of *gfp* in UTGFP1 and the region deleted in UTIΔ*neu* are both indicated.

To infect and persist within the urinary tract, UPEC must deploy a variety of appropriate virulence factors (reviewed in references 13 and 14). These factors permit the various stages in UTI to be achieved, including colonization, nutrient acquisition, adhesion to uroepithelial cells, invasion, intracellular replication, and subsequent spread (13). A key virulence factor associated with UPEC is the polysaccharide capsule, or K antigen, on the surface of the bacterial cell (15). E. coli capsules have been classified into four groups (groups 1 to 4) on the basis of a number of biochemical and genetic criteria (16). The expression of group 2 capsules (typified by K1) is associated with invasive isolates of E. coli, including UPEC. The group 2 capsule gene cluster is composed of three regions (Fig. 1) and is regulated principally at the level of transcription. This involves the action of several global regulators (H-NS, SlyA, IHF, and RfaH) acting at two convergent temperature-regulated promoters (17–21). To date, temperature is the only known environmental signal regulating transcription from these promoters: transcription occurs at 37°C in the host but not at temperatures below 25°C outside the host (22). However, it is likely that other, as-yet-unidentified, host-induced stimuli feed into this regulatory network.

It has been known for some time that for invasive bacterial pathogens the expression of the K antigen is important in allowing bacteria to combat innate host defenses such as phagocytosis and resistance to complement-mediated killing (23, 24). However, it is now clear that polysaccharide capsules play additional, more subtle roles in mediating interactions between the pathogen and the host (25). Such roles include moderating induction of chemokines and cytokines, interacting with Toll-like receptors (TLRs), perturbation of mucus clearance, and intracellular survival. Indeed, it has been shown that a lack of K1 capsule severely disrupted the development and progression of IBC (26). In contrast to the wild-type strain, which generates high-density IBC, mutants defective in capsule expression formed low-density groupings of intracellular bacteria (26). Clearly, these data indicate a requirement for capsule expression for efficient IBC formation. However, during the initial stages of UTI, during growth in urine, it has been shown that urinary tract isolate CFT073 and asymptomatic bacteriuria (the occurrence of bacterial growth in urine without symptoms) strain 83972 both downregulate capsule gene expression (27, 28).

It has been shown with a number of pathogens that capsule expression interferes with the attachment of bacteria to target cells (29–31). Therefore, an attractive hypothesis would be that UPEC downregulates expression of its capsule genes in urine to allow optimum adhesion to epithelial cells. Implicit in this is that upon invasion of uroepithelial cells, there has to be a transition from low or no capsule expression to increased capsule expression in order to allow IBC formation, indicating that the capsule may be spatially and temporally regulated during UTI. Capsule expression has for many other bacterial pathogens reported to be phase variable, thus giving the bacterial populations phenotypic diversity allowing them to quickly adapt to constantly changing environments (32–34). Although a phase-variable transacetylase exists that can result in capsular sialic acid variation in E. coli, there is no evidence that capsule expression itself is subject to phase variation (35).

In the present study, we sought to determine if there is downregulation of the K1 capsule genes in clinical UPEC isolate UTI89 during growth in urine. As part of these studies, we discovered that as well as being downregulated in urine, the capsule genes appear to be expressed in a nonuniform manner, resulting in phenotypic diversity of the capsule on the cell surface. We identified the existence on a distinct unencapsulated subtype in the clonal population and provide evidence that these capsule-negative bacteria are the initial colonizers of bladder epithelial cells. We propose that this physiological diversity in the capsule ensures that at least some of the bacterial cells have the appropriate phenotype in a given environment, increasing the strain's fitness during UTI.

## MATERIALS AND METHODS

### Bacterial strains and culture conditions.

The bacterial strains and plasmids used in this study are listed in Table 1. Bacteria were routinely grown in Luria-Bertani (LB) medium at 37°C and supplemented with antibiotics as appropriate at the following concentration: 50 μg/ml for kanamycin, 100 μg/ml for ampicillin, and 25 μg/ml for chloramphenicol. For growth in urine, urine was collected from healthy female volunteers who had no history of UTI or antibiotic use within the last 2 months, filter sterilized, and refrigerated for use within a week. Pools from three to five volunteers (pH 6.4 to 6.8; 350 to 400 mOsm/kg of water) were used for each experiment. When carrying out comparisons between urine-grown and LB medium-grown bacteria, cells were grown to approximately the same point in the growth curves in each medium. It should be noted that UTI89 grows to a much lower optical density at 600 nm (OD_600_) in urine than in LB medium (see Fig. S1 in the supplemental material). For infection experiments, bacteria were grown statically in LB medium to an OD of 0.5.

**TABLE 1:** Strains and plasmids used in this study

Strain or plasmid	Relevant genotype or feature	Reference or source
Strains		
UTI89	Clinical cystitis isolate	52
UTGFP1	UTI89 PR1::*gfp*, *gfp* between *kpsE* and *kpsF*	This study (Fig. 1)
UTIΔ*neu*	UTI89 Δ*neuDBACES*	This study (Fig. 1)
UTGFP1red	UTGFP1 × pBRdsRED	This study
Plasmids		
pBR322	Amp^r^ Tet^r^	53
pBRdsRED	pBR322 Tet::dsRED, Amp^r^	This study
pCoC2	*gfp*^TCD^ FRT-Kan^r^-FRT	38
pDOC-C	Sce1-Kan^r^-Sce1 *sacB* Amp^r^	37
pACBSce1	I-Sce1, λ-Red, Cml^r^	37
pCP20	FLP Amp^r^	39
pKT274	K1 capsule biosynthesis, Amp^r^	54

### Cell line and bacterial infections.

The pediatric human bladder cell line PD07i (36) was cultured in serum-free EpiLife medium (Invitrogen) supplemented with 1% human keratinocyte growth supplement (Invitrogen). For quantifying initial adherence and invasion, PD07i cells were seeded into 6-well culture dishes and after overnight propagation were infected with bacteria in fresh EpiLife medium (or pooled urine) (multiplicity of infection [MOI] = 100), centrifuged for 4 min at 500 × *g* to synchronize infection, and incubated for 2 h at 37°C and 5% CO_2_. Following incubation, cells were washed four times with phosphate-buffered saline (PBS) and lysed with 0.5% Triton X-100 in PBS (*T*_0_), and serial dilutions of bacteria were plated on LB agar. For microscopy, PD07i cells were seeded into 6-well culture dishes onto sterile glass coverslips and infected as described above. Following PBS washes, fresh medium containing 100 μg/ml of gentamicin was added to kill extracellular bacteria and the cells were returned to the incubator until the time point required.

### Construction of PR1-*gfp* reporter strain UTGFP1.

We used the gene doctoring method of Lee et al. (37) to construct a transcriptional fusion in which *gfp* is under the control of the capsule gene promoter PR1, at the capsule locus between *kpsF* and *kpsE*, on the chromosome of UTI89 (Fig. 1). Briefly, plasmid pCoC2 (38) was used as a template to amplify *gfp-kan* using primers PR1*gfp*-F (5′-TCGAGCGGATCCATTTTCGACTAATAAGCAACGGGGTTCGAGAGGTTAGTCTATGAGCAAAGGCGAAGAGCTGTTCACCGGTG-3′) and PR1*gfp*-R (5′-TCGAGCGTCGACGTAACGATTATCAAAGTTTCAGTTGGGGCGCAAACTCAATGAGCTCAGTCGAAAGACTGGGCCTTTCGCCC-3′). The amplicon (∼2.24 kb) was digested with BamHI and SalI and ligated to the corresponding sites in plasmid pDOC-C. The resulting plasmid was transformed into UTI89 alongside pACBSce1. Transformants were patched on 5% sucrose before induction of recombination onto the chromosome as described previously (37). The location of *gfp-kan* on the UTI89 chromosome was confirmed by PCR. The kanamycin cassette was then removed using plasmid pCP20 as described previously (39) and the resulting strain, UTGFP1, was confirmed by PCR and sequencing.

### Construction of capsule deletion strain UTIΔ*neu*.

The capsule-negative strain UTIΔ*neu* was also constructed using the gene doctoring method (37). Primers Δ*neu*-F (5′-TCGAGCGGATCCCTACTTTAAGATTTAATTTTACGACTGGTACTGTAATAGAATATAAAATGTAGCTTTTAGCCCGGGTGTAGGCTGGAGCTGC-3′) and Δ*neu*-R (5′-TCGAGCGTCGACCCTATAGTGGTTACATTCCAATATTATGCCTTGGAAATATTTAACTGAGACATATCATATGAATATCCTCCTTAGTTC-3′) containing the flanks of region 2 were used to amplify the Kan cassette from plasmid pCoC2, and cloning, recombination onto the chromosome, and subsequent removal of the Kan cassette were carried out as described above.

### Immunofluorescent microscopy. (i) Bacterial cells.

Bacterial cells grown to mid-log phase in either LB medium or urine were washed three times in PBS and resuspended in PBS before spotting of 10 μl on microscope slides. Cells were allowed to air dry before heat fixation. For staining with anti-K1 antibody (Ab) [a monoclonal IgG2A Ab raised against meningococcal group B and E. coli K1 capsular polysaccharides which are identical homopolymers of α(2→8)-linked sialic acid (40)], fixed samples were blocked in 1% bovine serum albumin in PBS (BSA-PBS), washed three times with PBS, and incubated with anti-K1 Ab (1/400 dilution of a 4-mg/ml stock in 1% BSA-PBS) for 20 min at room temperature (RT). Samples were then washed three times with PBS and incubated with secondary Ab (1/500 dilution donkey polyclonal anti-mouse IgG conjugated with Alexa Fluor 594 [Abcam]) and 50 mM 4′,6-diamidino-2-phenylindole (DAPI) for 20 min at room temperature. Slides were washed again three times with PBS and mounted with Mowiol (Sigma).

### (ii) Infected PD07i cells.

Infected bladder cells were washed four times with PBS and then, to stain extracellular bacteria only, incubated with a polyclonal rabbit anti-E. coli IgG antibody (Thermo Scientific) at a 1/200 dilution in EpiLife-10% goat serum (Sigma) for 30 min at 37°C and 5% CO_2_. Samples were washed three times in PBS and then fixed in 3% paraformaldehyde. To permeabilize the bladder cell plasma membrane, samples were incubated in 0.1% Triton X-100 in PBS for 4 min. The capsule was then stained using anti-K1 Ab and donkey anti-mouse Alexa Fluor 594 (secondary Ab to anti-K1) as described above. Donkey anti-rabbit IgG Cy5 (1/500 dilution [Abcam]) and DAPI were also added to the secondary Ab mixture to stain the anti E. coli Ab and cell nuclei, respectively. Samples were washed three times with PBS between each step and mounted with Mowiol.

Images were collected on an Olympus BX51 upright microscope using a 60×/0.30 Plan Fln objective and captured using a Coolsnap ES camera (Photometrics) through MetaVue software (Molecular Devices). Images were processed using ImageJ software (41).

### Flow cytometry analysis.

Bacteria were grown to the desired OD. Around 10^8^ cells were washed three times in PBS, blocked for 1 h in 1% BSA-PBS, and treated with anti-K1 Ab (1/400 dilution) in buffer A (1% BSA-PBS, 0.05% Tween 20) for 1 h at RT. After one wash with buffer A, bacteria were treated with secondary Ab (1/500 dilution of donkey anti-mouse Ab conjugated with Alexa Fluor 594) in buffer A for 1 h at RT and then washed three times in buffer A. A total of 100,000 cells were analyzed for fluorescence per sample using a BDFortessa flow cytometer.

### FACS and sample regrowth.

Bacterial cells were prepared for fluorescence-activated cell sorting (FACS) as described above. Cells were sorted into negative and positive subpopulations, each containing 50,000 cells. A total of 1,000 cells from each sort were reanalyzed by FACS to confirm proper sorting of the two populations. Cells were sorted straight into 1 ml of LB medium and grown at 37°C for 16 h, after which time 10^8^ cells from each culture were prepared for flow cytometry as described above.

### Statistical analyses.

*P* values were determined using two-tailed unpaired *t* tests, and normal distribution of samples was confirmed using the Shapiro-Wilk normality test in R (42). Data were considered significantly different at a *P* value of <0.05.

## RESULTS AND DISCUSSION

### Capsule expression in pooled human urine.

**FIG 2:**
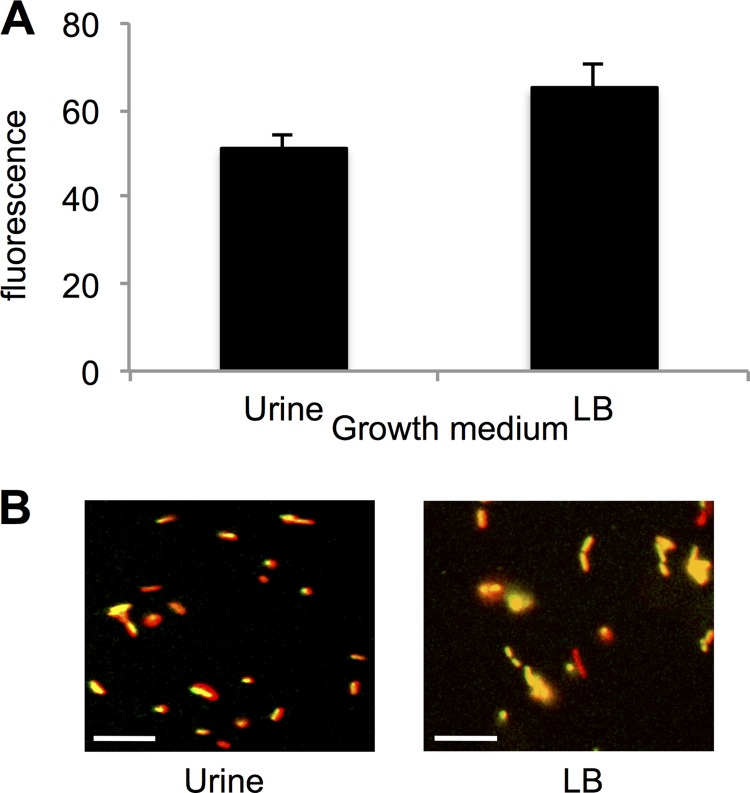
Downregulation of capsule genes in urine. Strain UTGFP1red was grown to mid-exponential phase in urine or LB medium, and *gfp* expression measured on a fluorescent plate reader (A) or visualized by fluorescence microscopy (B). Data are expressed as means ± standard deviations. *P* = 0.036 (*n* = 3). Cells expressing *gfp* appear yellow. A representative field of view, from over 50 visualized, is shown. Scale bars = 10 μm.

**FIG 3:**
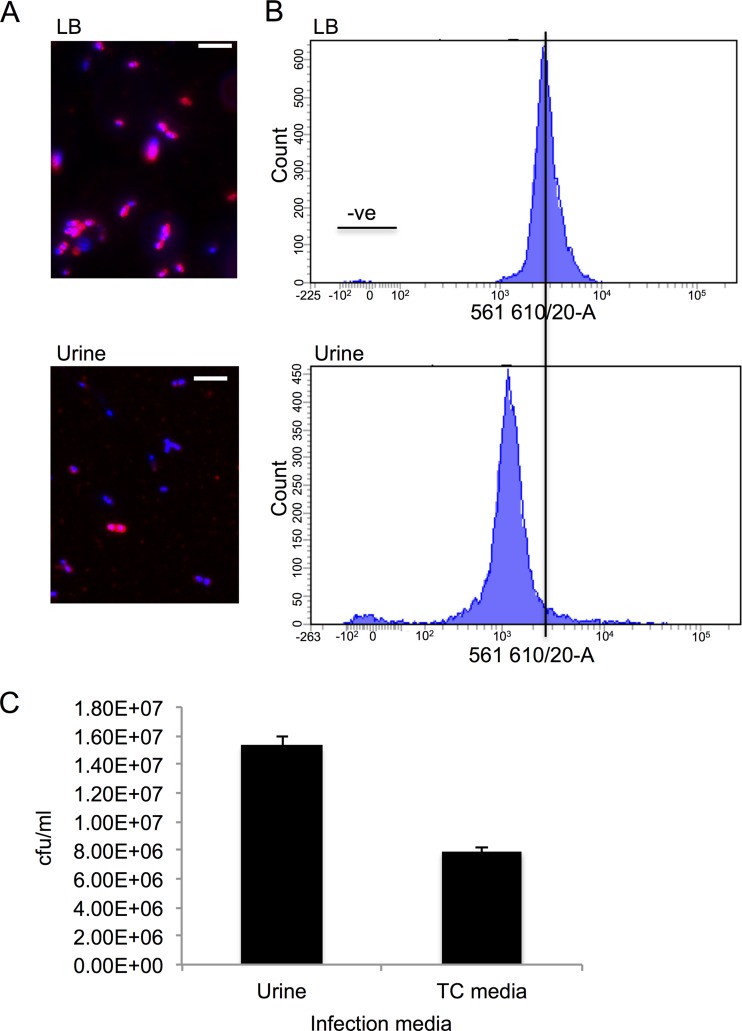
Decrease in amount of capsule on bacterial cell surface during growth in urine and evidence of an unencapsulated subpopulation. (A) UTI89 was grown to mid-exponential phase to late exponential phase in urine (OD_600_ = 3.0) or LB medium (OD_600_ = 1.0). Samples were fixed on microscope slides and stained with DAPI, anti-K1 primary Ab, and Alexa Fluor 594-conjugated secondary Ab. Scale bars = 10 μm. Bacteria expressing capsule appear pink, while unencapsulated bacteria appear blue. Over 50 fields of view for each sample were visualized, and a representative field of view is shown. (B) UTI89 was grown and stained with the same Abs as mentioned above before analysis by flow cytometry. The *x* axis shows fluorescence intensity, and the *y* axis shows the number of cells. The small unlabeled population in LB medium is indicated (-ve). (C) PD07i cells were infected with UTI89 for 2 h in either tissue culture (TC) medium or urine. To measure initial adhesion and invasion, cells were lysed and cell numbers determined (*n* = 4). *P* < 0.001.

To test the hypothesis that capsule gene transcription is downregulated during growth in urine compared to growth in LB, we constructed a reporter strain (UTGFP1) in which *gfp* is under the control of the capsule gene promoter PR1 (Fig. 1). Our results show a slight but significant decrease in *gfp* expression in strain UTGFP1 (Fig. 2A) when grown in pooled human urine, indicating that the capsule genes, under the control of PR1, are indeed downregulated during growth in urine. However, when strain UTGFP1red (Table 1) was grown to mid-exponential phase to late exponential phase in LB medium and urine and examined at the single-cell level, heterogeneity in *gfp* expression could clearly be seen (Fig. 2B), with some cells appearing to express no *gfp* at all. Variability in expression from this promoter has been observed previously (26). Morphometric analysis of 100 similar-size individual cells in these populations confirmed an overall decrease in green fluorescence intensity in the urine-grown sample (see Fig. S2 in the supplemental material). To examine if this downregulation and heterogeneity in gene expression were translated to the cell surface, wild-type UTI89 was grown in both LB medium and urine and then cells were examined by immunofluorescence microscopy using an anti-K1 Ab (40). The results clearly show (Fig. 3A) less capsular material on the surface of urine-grown bacteria. However, in both media, an unencapsulated subpopulation of bacteria was evident. Flow cytometry analyses of LB medium- and urine-grown bacteria confirmed less capsular material present on the urine-grown cells (the shift in fluorescence is less) and also confirmed the existence of an unlabeled and hence unencapsulated subpopulation in both media, with this population being approximately 3 times more abundant in urine-grown UPEC (Fig. 3B). Approximately 0.8% of the population appeared to be capsule negative in LB medium, while in urine this population increased to 2.4%. The existence of these unencapsulated populations was confirmed by infecting LB medium-grown and urine-grown UTI89 with K1-specific bacteriophage and enumerating the phage-resistant (and hence unencapsulated) bacteria (see Fig. S3 in the supplemental material). This downregulation of capsule in urine may allow more bacteria to efficiently adhere to the host urothelium; however, it can be seen that many cells still produce at least some capsule in urine, and presumably these bacteria will remain shielded from the antimicrobial peptides and proteins known to be present in this environment (43). This observed phenotypic diversity therefore might be allowing the “best of both worlds” for the bacterium, that is, an encapsulated subset to carry on dividing in urine while an unencapsulated population can invade the host.

In order to test the hypothesis that downregulation of capsule in urine allows increased adherence of UPEC to the urothelium, we carried out an infection assay with UTI89 and PD07i bladder epithelial cells in pooled human urine and compared adhesion and invasion at 0 h after end of log-phase growth to an assay in standard tissue culture medium. We found that when the infection was carried out in urine (as opposed to tissue culture medium), nearly double the amount of UTI89 initially adhered to and invaded the bladder cells (Fig. 3C). Preincubation of the bladder epithelial cells in urine (for 2 h) before carrying out the infection in tissue culture medium had no change in adherence and invasion, indicating that the effect was due to a change in the bacterial population and not due to a change in the epithelial cells (data not shown). Of course, this initial increase in adherence and invasion may have been due to changes, other than capsule, on the bacterial cell surface. For example, an increase in type 1 fimbriae in urine-incubated cells may also lead to the same result. However, urine-incubated bacteria showed less mannose-sensitive agglutination of guinea pig erythrocytes than tissue culture medium-incubated bacteria, indicating that the observed increase in adherence and invasion was not due to greater expression of type1 fimbriae in urine-incubated UPEC (see Fig. S4 in the supplemental material). It should be noted that in this experiment no mannose-resistant agglutination was detectable following incubation in either urine or tissue culture medium (see Fig. S4). This supports the notion that the differences in adherence and invasion cannot be readily explained by increased fimbria expression. These results indicate that downregulation of capsule in urine-grown cells may, at least in part, be responsible for increased adherence to and invasion of bladder epithelial cells.

It should be noted that transcriptomic studies on UPEC in voided urine from individuals experiencing symptomatic UTI do show an increase in *kps* genes (44, 45). Such urine will contain a large number of bacteria that have already entered the IBC pathogenic pathway (12) and therefore, according to previous studies, will already be expressing capsule genes (26).

### Existence of unencapsulated subpopulations is not due to a genetic change.

**FIG 4:**
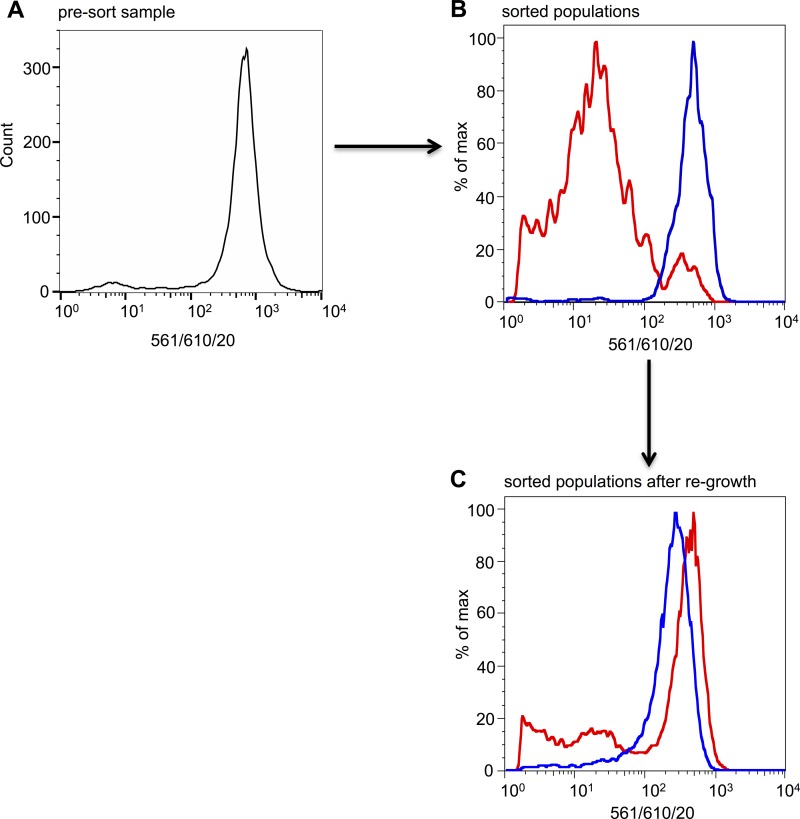
The unencapsulated population is not due to a heritable genetic change. UTI89 was grown to late exponential phase and then labeled with the anti-K1 Ab and Alexa Fluor 594-conjugated secondary Ab before being sorted by FACS (A). A total of 50,000 unencapsulated bacteria and 50,000 encapsulated bacteria were sorted. A total of 1,000 cells from each sort were then reanalyzed to check sort accuracy (B). Negative (or unlabeled) cells correspond to the red population, while the positive (or labeled) population is shown in blue. After overnight growth of the sorted populations, the two cultures were relabeled as before and analyzed by flow cytometry (C).

We hypothesized that if the capsule-negative population was the result of a genetic change or spontaneous mutation, such a change would persist in future generations so that unencapsulated sorted cells would remain unencapsulated when regrown, whereas if the change was due to phenotypic variation, then unencapsulated sorted bacteria should be able to differentiate into two populations again when regrown. To test this, bacteria were sorted into negative (unencapsulated) and positive (capsulated) populations based on the binding of the anti-K1 Ab, regrown overnight, and analyzed again by flow cytometry after Ab labeling (Fig. 4). The results show that after regrowth the negative population shifted to a mostly capsule-positive population, indicating that the existence of this negative subpopulation was a reversible trait. The two populations grew at the same rate during regrowth, so the population shift was not a direct result of the small positive population in the negative sort having a higher growth rate (data not shown). In order to be sure that the two populations did not arise due to a transient genetic rearrangement, as is the case in bacterial phase variation, the PR1 and PR3 regions (Fig. 1) from both populations were amplified by PCR and sequenced. The sequences of both promoter regions in both populations were identical to the wild-type sequence, confirming that the phenotypic variation was not due to a genetic change (data not shown).

Conventional analyses of clonal populations often assume all bacteria are identical. Consequently, at the capsule-permissive temperature of 37°C, all of the cells in the population will express a capsule (22). However, this study shows, for the first time, that at 37°C not all cells produce capsule. The mechanism behind this heterogeneity is, at present, unclear. Phenotypic variation not caused by a genetic change in DNA sequence has been observed in various bacterial systems (46, 47) and is primarily a result of stochastic fluctuations in cellular components, or “noise.” The origins and consequences of molecular-level noise for gene expression have been well documented (48). In certain cases amplification of noise can cause the population to bifurcate into two phenotypic distinct subpopulations, a phenomenon known as bistability.

### Capsule negatively affects adhesion of UTI89 to a bladder epithelial cell line.

**FIG 5:**
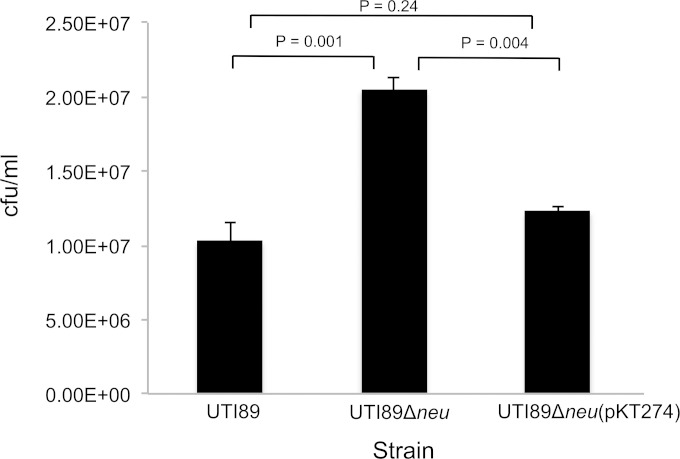
K1-negative strain shows greater initial adhesion to and invasion of PD07i cells. PDO7i cells were infected with UTI89, UTIΔ*neu*, or UTIΔ*neu*(pKT724) as described in Materials and Methods. After 2 h, bladder cells were washed thoroughly to remove nonadherent bacteria and then lysed, and adherent and intracellular bacteria were enumerated. Data are expressed as means ± standard deviations. *n* = 4. *P* values are shown above the corresponding strains.

The expression of a bacterial polysaccharide capsule has been shown to inhibit intimate adhesion to epithelial cells in a number of bacterial strains (29–31). If this was the case with UTI89, then we would predict a capsule-negative mutant to show increased adhesion to and invasion of bladder epithelial cells. To address this, we constructed a capsule-null mutant, UTIΔ*neu* (Table 1), that has a deletion in the region 2 synthesis genes (Fig. 1). Resistance to the K1-specific bacteriophage and inability to react with anti-K1 Ab confirmed the lack of capsule production by this strain (data not shown). We then tested the ability of UTIΔ*neu* to adhere to and invade the bladder epithelial cell line PD07i. Figure 5 shows that there is a significant increase in adherence and invasion at *T*_0_ with UTIΔ*neu* compared to wild-type UTI89. The Δ*neu* mutation could be complemented by supplying the capsule genes in *trans* on plasmid pKT274 (49). These results confirm that the K1 capsule of UTI89 can negatively affect adhesion of the bacteria to the human bladder epithelial cell line PD07i. This is in contrast to the experiments of Anderson et al. (26) that showed no significant difference in initial adherence to and invasion of mouse bladders by an unencapsulated UTI89 mutant. Specific differences in the expression of genes encoding adherence factors of UPEC have been observed during human UTI and murine experimental UTI (44). These differences may account for the differences in adherence and invasion we observed with the K1-negative strain and the human PD07i cell line compared to those in the murine study of Anderson et al.

### The unencapsulated subpopulation is the initial colonizer of bladder epithelial cells.

**FIG 6:**
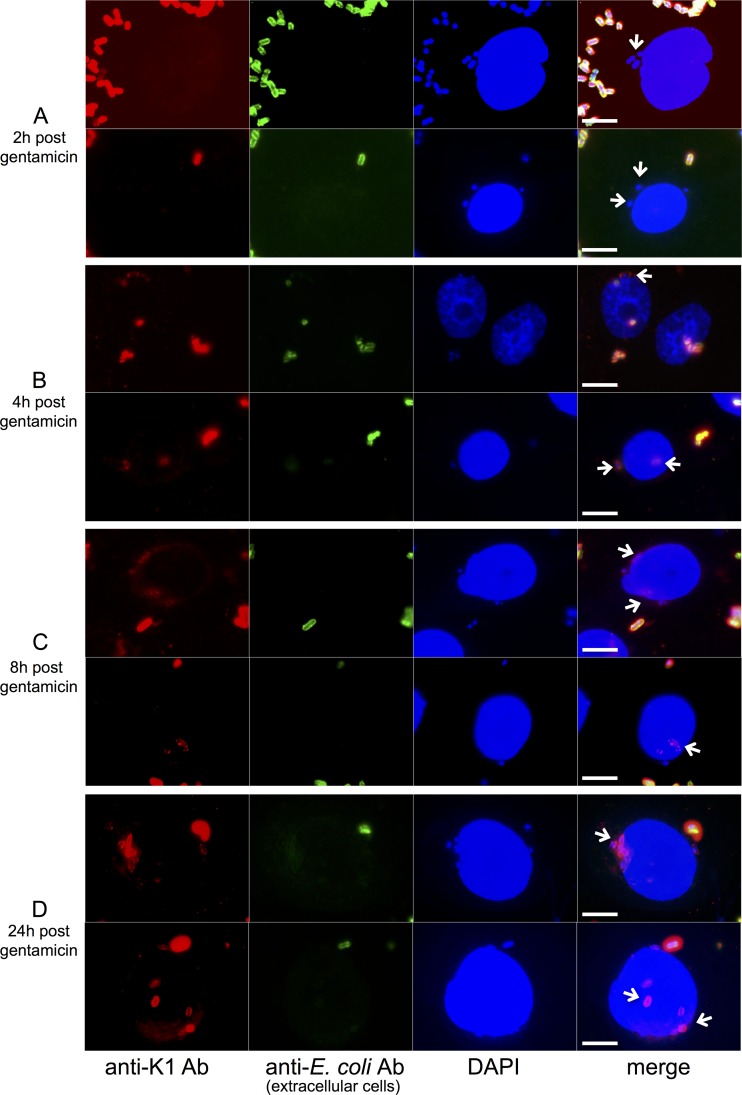
Unencapsulated UTI89 preferentially adhere to and invade PD07i cells before expressing capsule. PD07i cells were infected with UTI89 for 2 h as described in Materials and Methods. At the time points indicated following gentamicin treatment (to kill extracellular bacteria), cells were incubated with anti-E. coli Ab before fixation to stain extracellular bacteria. Then cells were permeabilized and stained with anti-K1 Ab, DAPI, and secondary Abs as described in Materials and Methods. Cells were examined by fluorescence microscopy. A montage of the individual fluorescent channels is shown alongside the composite image. The intracellular bacteria are most often visualized in the perinuclear region of the PD07i cells, which has been noted previously (50). Scale bar = 10 μm. Arrows highlight some of the intracellular bacteria. The experiment was carried out four times, with more than 20 fields of view examined for each time point. Two representative fields of view are shown for each time point.

Since we have shown that there exists an unencapsulated subpopulation in the clonal UTI89 population and that capsule can inhibit adherence to and invasion of epithelial cells, we surmised that these unencapsulated UTI89 organisms might be the initial bacteria to colonize and invade bladder epithelial cells. To test this, we carried out a gentamicin protection experiment in which PD07i cells were infected with wild-type UTI89 for 2 h, followed by gentamicin treatment to kill extracellular bacteria. Cells were then stained with anti-K1 Ab and examined by immunofluorescence microscopy at the time points indicated in Fig. 6. To distinguish between intracellular and extracellular bacteria, samples were first stained with an anti-E. coli Ab before fixation and permeabilization of the bladder cell membrane. Thus, intracellular bacteria were protected from labeling and only extracellular bacteria were stained with this Ab. At 2 h after gentamicin treatment, all intracellular bacteria appeared to have no capsule or capsular material on their cell surface, whereas extracellular bacteria appeared largely encapsulated (Fig. 6A). This indicates that the unencapsulated bacteria in the population do appear to be the initial bacteria to attach to and invade the epithelial cells. Two hours later, at 4 h after gentamicin treatment, capsular material could be detected on some intracellular bacteria (Fig. 6B), and by 8 h after gentamicin treatment, completely encapsulated bacteria could be visualized inside the epithelial cells (Fig. 6C). At 24 h after gentamicin treatment, many of the intracellular bacteria were encapsulated (Fig. 6D). These results indicate that unencapsulated bacteria preferentially bind and invade the bladder epithelial cells and that once internalized, at least some of the bacteria upregulate expression of the capsule genes. This is in agreement with the work of Berry et al. (50), who showed upregulation of *kpsD* (by quantitative reverse transcription-PCR) in the intracellular environment of PD07i cells. Also, since we know that the capsule is important for IBC formation, we would predict upregulation of these genes in the intracellular environment (26). Recently, Baum et al. (51) showed upregulation of capsule genes in response to PafR, a transcriptional regulator that appears to be expressed only *in vivo*. PafR therefore may be responsible, at least in part, for the intracellular increase in capsule production. Interestingly, PafR was not expressed during growth in urine (51); therefore, the effect we see on capsule expression following growth in urine cannot be explained by PafR regulation.

In summary, we have shown there is downregulation of capsule genes and a decrease on surface K1 capsule in urine-grown UPEC compared to those in the organism grown in LB medium. We have also shown that capsule expression is heterogeneous, with distinct unencapsulated and encapsulated subtypes being present in the clonal population. The unencapsulated population appears to preferentially bind and invade bladder epithelial cells, after which intracellular bacteria can start to produce capsule, presumably to aid in intracellular survival. We propose that UPEC exploits this heterogeneous phenotype to deal with the constantly changing environment it will encounter during UTI, and therefore, this diversity is a key factor in the microorganism's fitness.

## Supplementary Material

Supplemental material
