# Supplementary material for: Phenotypic Heterogeneity in Expression of the K1 Polysaccharide Capsule of Uropathogenic Escherichia coli and Downregulation of the Capsule Genes during Growth in Urine
Source: Infect Immun. 2015 Jun 15;83(7):2605–13. doi: 10.1128/IAI.00188-15 (PMC4468546; doi:10.1128/IAI.00188-15)
Supplement: Supplemental material [file supp_83_7_2605__index.html]

Phenotypic Heterogeneity in Expression of the K1 Polysaccharide Capsule of Uropathogenic Escherichia coli and Downregulation of the Capsule Genes during Growth in Urine — Supplemental material 

# Phenotypic Heterogeneity in Expression of the K1 Polysaccharide Capsule of Uropathogenic Escherichia coli and Downregulation of the Capsule Genes during Growth in Urine

## Supplemental material

- Supplemental file 1 -

  Fig. S1. UTI89 grows to a lower OD600 in urine than in LB.

  PDF, 76K
- Supplemental file 2 -

  Fig. S2. Downregulation of PR1-*gfp* in urine-grown UTGFP1red.

  PDF, 75K
- Supplemental file 3 -

  Fig. S3. Evidence for the existence of an unencapsulated population in LB- and urine-grown UTI89.

  PDF, 70K
- Supplemental file 4 -

  Fig. S4. Tissue culture medium-incubated UTI89 shows more mannose-sensitive hemagglutination than urine-incubated UTI89.

  PDF, 897K
