# Supplementary material for: Phenotypic Heterogeneity in Expression of the K1 Polysaccharide Capsule of Uropathogenic Escherichia coli and Downregulation of the Capsule Genes during Growth in Urine
Source: Infect Immun. 2015 Jun 15;83(7):2605–13. doi: 10.1128/IAI.00188-15 (PMC4468546; doi:10.1128/IAI.00188-15)
Supplement: Supplemental material [file IAI.00188-15_zii999091268so1.pdf]

Fig. S1

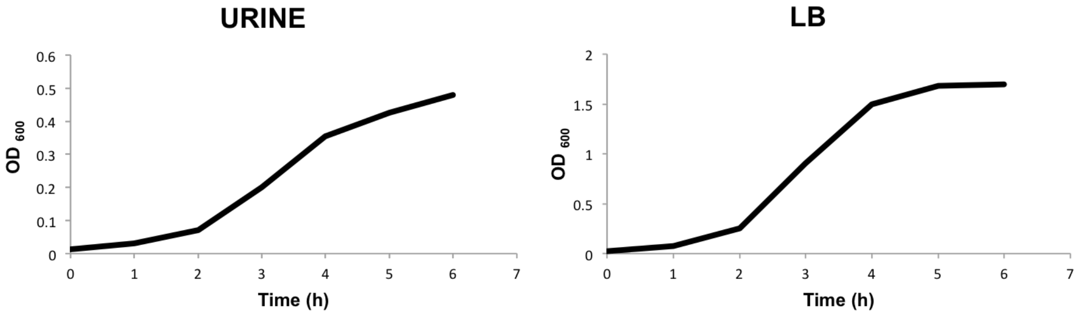

Fig. S1 UTI89 grows to a lower OD_{600} in urine compared to growth in LB. Overnight cultures of UTI89 grown in either urine or LB were diluted 1:100 in the same media and were grown shaking at 37°C. The OD_{600} was measured at the time points indicated. Growth curves are representative of at least three independent experiments.
