# Supplementary material for: Phenotypic Heterogeneity in Expression of the K1 Polysaccharide Capsule of Uropathogenic Escherichia coli and Downregulation of the Capsule Genes during Growth in Urine
Source: Infect Immun. 2015 Jun 15;83(7):2605–13. doi: 10.1128/IAI.00188-15 (PMC4468546; doi:10.1128/IAI.00188-15)
Supplement: Supplemental material [file IAI.00188-15_zii999091268so2.pdf]

Fig. S2

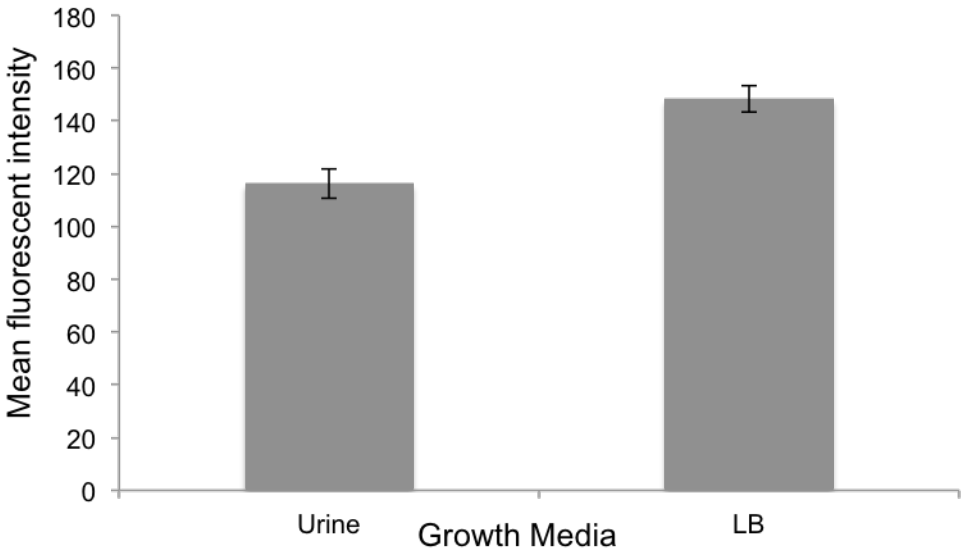

Fig. S2 Down regulation of PR1-*gfp* in urine-grown UTGFP1red. Morphometric analysis (using Image J software) of 100 individual similar sized bacterial cells, grown in either LB or urine to the same point in the growth curve, confirms an overall reduction in green fluorescence intensity and hence *gfp* expression in urine. $P<0.0001$ ($n=100$). Error bars represent the standard errors of the mean.
