# Supplementary material for: Phenotypic Heterogeneity in Expression of the K1 Polysaccharide Capsule of Uropathogenic Escherichia coli and Downregulation of the Capsule Genes during Growth in Urine
Source: Infect Immun. 2015 Jun 15;83(7):2605–13. doi: 10.1128/IAI.00188-15 (PMC4468546; doi:10.1128/IAI.00188-15)
Supplement: Supplemental material [file IAI.00188-15_zii999091268so3.pdf]

Fig. S3

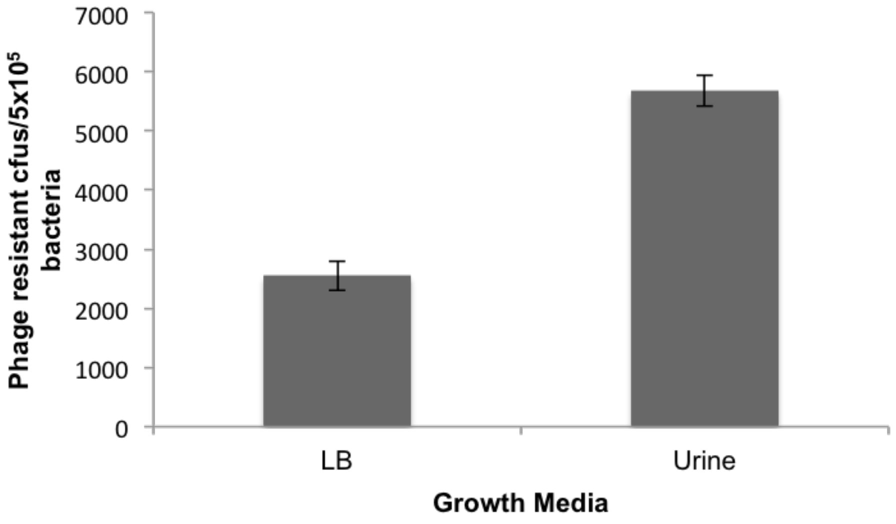

Fig. S3 Evidence for the existence of an un-encapsulated population in LB and urine grown UTI89. UTI89 was grown to mid-late log phase in LB and urine; approximately $5x10^5$ cells were incubated for half an hour at room temperature in 10 mM $MgSO_4$ with K1 bacteriophage (MOI=10). After incubation dilutions were plated on LB and the number of phage resistant bacteria enumerated. P<0.00001 (n=6). Values depict the means of independent experiments ± the standard errors of the mean.
