# Supplementary material for: Phenotypic Heterogeneity in Expression of the K1 Polysaccharide Capsule of Uropathogenic Escherichia coli and Downregulation of the Capsule Genes during Growth in Urine
Source: Infect Immun. 2015 Jun 15;83(7):2605–13. doi: 10.1128/IAI.00188-15 (PMC4468546; doi:10.1128/IAI.00188-15)
Supplement: Supplemental material [file IAI.00188-15_zii999091268so4.pdf]

Fig. S4

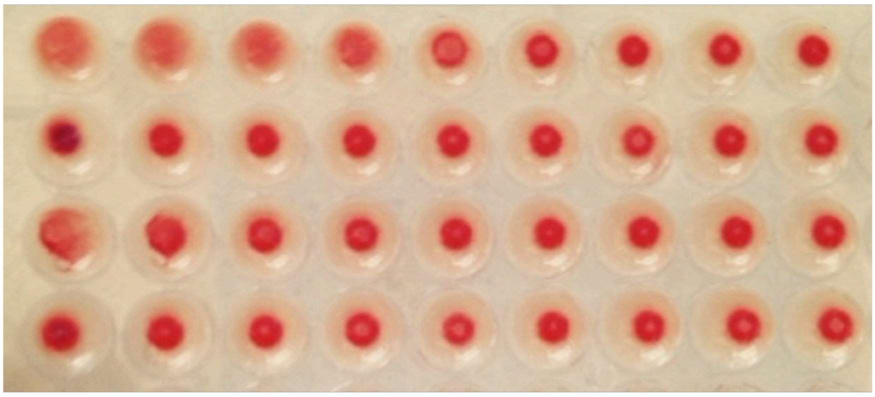

Fig. S4 Tissue culture media - incubated UTI89 show more mannose sensitive haemagglutination that urine - incubated UTI89. UTI89 grown to mid log phase were incubated for 2 h (37°C, 5% CO_2) in either tissue culture media or pooled human urine. Bacterial cells were then harvested, washed x 3 in PBS and re-suspended in PBS to OD = 2. Two fold serial dilutions of the bacteria were incubated with an equal volume of guinea pig erythrocytes, in the presence or absence of 50 mM D-mannose, in round bottom 96 well plates Agglutination can be seen as a diffuse matt of cells, whereas no agglutination results in a discrete button of red blood cells. Haemagglutination can clearly be seen in the tissue culture media-incubated cells with an end point dilution of 1/32. This agglutination is completely inhibited by mannose
